# Aterosclerose e Inflamação: Ainda Muito Caminho a Percorrer

**DOI:** 10.36660/abc.20200219

**Published:** 2020-05-12

**Authors:** Ricardo Wang, Bruno Ramos Nascimento, Fernando Carvalho Neuenschwander

**Affiliations:** 1 Universidade Federal de Minas Gerais Departamento de Cardiologia Intervencionista Belo Horizonte MG Brasil Universidade Federal de Minas Gerais, Departamento de Cardiologia Intervencionista, Belo Horizonte, MG – Brasil; 2 Hospital Vera Cruz Departamento de Cardiologia Intervencionista Belo Horizonte MG Brasil Hospital Vera Cruz, Departamento de Cardiologia Intervencionista, Belo Horizonte, MG – Brasil; 3 Hospital UNIMEDBH Belo Horizonte MG Brasil Hospital da UNIMEDBH, Belo Horizonte, MG - Brasil

**Keywords:** Aterosclerose, Inflamação, Interleucina-18, Trombose, Doença da Artéria Coronária

Desde os trabalhos iniciais de Russell Ross^1^ sobre a importância da inflamação no desenvolvimento, na instabilização e ruptura da placa aterosclerótica, que podem resultar na síndrome coronariana aguda (SCA) ou acidente vascular encefálico, houve crescimento das publicações sobre as vias de ativação, ampliação e perpetuação do processo inflamatório, que vai além do simples entendimento fisiopatológico, mas principalmente em encontrar oportunidades de tratamentos específicos, com intuito de diminuir o chamado “risco residual” (eventos cardiovasculares que ocorrem em pacientes mesmo com níveis de LDL colesterol dentro das metas terapêuticas).^2^ Estima-se que aproximadamente 100.000 casos novos de infarto agudo do miocárdio ocorrem por ano no Brasil,^3^ segundo dados do DATASUS, se consideramos taxas de mortalidade de 5-10%, é de se esperar que aproximadamente 90.000 pacientes por ano vão para prevenção secundária. Se todos os pacientes receberem dose máxima de estatina, mesmo assim, em torno de 40%^2^ ainda apresentam risco residual de eventos, isto é, 36.000 doentes são de alto risco para novos eventos cardiovasculares.

Nesta edição, foi publicado um estudo^4^ sobre o papel da Interleucina-18 (IL-18) e proteína precursora da trombina (TpP) na SCA. A TpP é um marcador da ativação do sistema de coagulação, e neste estudo os autores observaram elevação desta proteína nos pacientes com quadro de SCA, fato que corrobora com a importância do sistema de coagulação neste cenário.^5 , 6^ Estudos anatomopatológicos de placas demonstraram que a ruptura da capa fibrótica nem sempre é acompanhada de uma SCA,^6^ para uma “tempestade perfeita” é necessário a associação de “placa vulnerável” com “sangue vulnerável” (estado de hipercoagulabilidade).^5^ Este estudo corrobora que a TpP pode ser um biomarcador útil para o diagnóstico de SCA.

As interleucinas são moléculas sinalizadora entre células do sistema inflamatório, podendo induzir, proliferar e perpetuar a inflamação, mas também podem modular e reduzir a inflamação. A IL-18 é produzida pelos macrófagos, em resposta a ao processo de fagocitose e produzido via sistema de caspases, age nas células Th1 estimulando a produção de interferon-g (INF-g) e Interleucina-1b,^7^ e sua elevação na aterosclerose está associada a ruptura de placa.^8^ Placas consideradas vulneráveis apresentam núcleo necrótico com presença de grande número de células inflamatórias, principalmente macrófagos. A inflamação age inibindo as células musculares lisas o que acarreta na formação de uma capa fibrosa mais fina, portanto mais propensa à ruptura.

Por ser um estudo transversal, temos a limitação em estabelecer nexo causal, IL-18 causou a SCA ou a SCA causou a elevação da IL-18? Neste contexto, a elevação da IL-18 pode ser secundária a necrose miocárdica como sugerido por Seta et al.,^9^ pois durante o processo de necrose miocárdica e reparo há ativação do sistema inflamatório, incluindo os macrófagos. Outra limitação deste estudo, é relacionado a amostra, que além de pequena, foi por conveniência, o que por si, pode ser uma fonte de viés. O grupo controle pode ser problemático, foi selecionado a partir de pacientes submetidos a coronariografia, esta sem evidências de lesão obstrutiva angiográfica (lembramos que por algum motivo estes foram submetidos a um procedimento invasivo). A coronariografia tem limitações em diagnosticar aterosclerose,^10^ pois na fase inicial da aterosclerose ocorre o efeito de remodelamento positivo (efeito Glagov); placas com 70% de área total do vaso, podem se apresentar na angiografia com lesão menos 30%.^11^ Portanto, a coronariografia não deveria ser utilizada como padrão ouro para afastar aterosclerose. Além disso, é sabido que as placas que mais provocam SCA são placas leves e inflamadas.^5^ Estas limitações poderiam explicar os níveis mais elevados (apesar de estatisticamente não significativos) de IL-18 no grupo controle em comparação com pacientes com doença coronariana estável neste estudo (663,25 pf/mL ± 993,93 versus 353,81 pg/mL± 273,65 respectivamente, p = NS).

O bloqueio da IL-18 deve ser usado na prevenção primária? Ou melhorar o remodelamento ventricular pós infarto? Dada a complexidade do sistema imunológico, e sua importância na contenção de processo infecciosos e no sistema de dano-reparo celular, o seu bloqueio pode ter consequências. No caso da IL-18, que é parte do sistema caspase, responsável pelo combate a infecções intracelulares, poderíamos observar o aumento de infecções tais como a tuberculose. No estudo Cantos (Antiinflammatory Therapy with Canakinumab for Atherosclerosis Disease), no qual o bloqueio da interleucina 1-b esteve associado com discreto aumento das taxas de infecção (não significativo), artrite e câncer fatal (estatisticamente significativo).^12^ Em relação ao remodelamento, os resultados são conflitantes, bloqueio da Interleucina 1b com anakira foi promissor,^13^ mas o bloqueio do fator de necrose tumoral gama aumentou a mortalidade. Em modelos experimentais, ao bloqueio da IL-18, se mostra promissor em melhorar o remodelamento pós infarto.^14^

Apesar do entusiasmo inicial com os resultados do uso de colchicina e canakinumab, os benefícios líquidos ainda são tímidos.^12 , 15^ Por isso da necessidade de investigar outras vias inflamatórias. Ainda temos mais perguntas do que respostas: Qual via bloquear? Quando (prevenção primária ou secundária)? E qual intensidade e por quanto tempo?
