# Supplementary material for: Aterosclerose e Inflamação: Ainda Muito Caminho a Percorrer
Source: Arq Bras Cardiol. 2020 May 12;114(4):699–700. [Article in Portuguese] doi: 10.36660/abc.20200219 (PMC9744348; doi:10.36660/abc.20200219)
Supplement: Translation

# Translation: Atherosclerosis and Inflammation: Still a Long Way to Go

**Authors:** Ricardo Wang, Bruno Ramos Nascimento, Fernando Carvalho Neuenschwander

**Affiliations:** 1 Brazil Universidade Federal de Minas Gerais, Departamento de Cardiologia Intervencionista, Belo Horizonte, MG – Brazil; 2 Brazil Hospital Vera Cruz, Departamento de Cardiologia Intervencionista, Belo Horizonte, MG – Brazil; 3 Brazil Hospital da UNIMEDBH, Belo Horizonte, MG - Brazil

**Keywords:** Atherosclerosis/physiopathology, Inflammation, Interleukin-18, Thrombosis, Coronary Artery Disease

Short Editorial related to the article: Role of Interleukin-18 and the Thrombus Precursor Protein in Coronary Artery Disease.

Ever since the initial works by Russell Ross^1^ on the importance of inflammation in the development, instability and rupture of the atherosclerotic plaque, which can result in acute coronary syndrome (ACS) or stroke, there has been an increase in publications on the activation pathways, expansion and perpetuation of the inflammatory process, which goes beyond the simple pathophysiological understanding, but mainly in finding specific treatment opportunities, aiming to reduce the so-called “residual risk” (cardiovascular events that occur in patients even when LDL cholesterol levels are within the therapeutic goals).^2^ It is estimated that approximately 100,000 new cases of acute myocardial infarction occur each year in Brazil,^3^ according to data from DATASUS. If we consider mortality rates of 5-10%, it is expected that approximately 90,000 patients per year will go to secondary prevention. If all patients receive a maximum statin dose, even so, approximately 40%2 will still have a residual risk of events, i.e., 36,000 patients will be at high risk for new cardiovascular events.

This issue brings a study^4^ on the role of Interleukin-18 (IL-18) and thrombin precursor protein (TpP) in ACS. TpP is a marker of the coagulation system activation and, in this study, the authors observed an increase in this protein in patients with ACS, a fact that corroborates the importance of the coagulation system in this scenario.^5 , 6^ Anatomopathological studies of plaques have shown that the fibrotic layer rupture is not always accompanied by ACS,^6^ as for a “perfect storm” to occur, it is necessary to associate a “vulnerable plaque” with “vulnerable blood” (hypercoagulable state).^5^ This study corroborates that TpP can be a useful biomarker for the diagnosis of ACS.

Interleukins are signaling molecules among cells of the inflammatory system, which can induce, proliferate and perpetuate inflammation, but they can also modulate and reduce inflammation. IL-18 is produced by macrophages, in response to the phagocytosis process and it is produced via the caspase system, acts on Th1 cells by stimulating the production of interferon-g (INF-g) and Interleukin1b,^7^ and its elevation in atherosclerosis is associated with plaque rupture.^8^ Plaques considered vulnerable have a necrotic nucleus with a large number of inflammatory cells, mainly macrophages. Inflammation acts by inhibiting smooth muscle cells, which leads to the formation of a thinner fibrous cap, therefore more prone to rupture.

As this is a cross-sectional study, there is a limitation in establishing a causal association; did IL-18 cause ACS or did ACS cause IL-18 elevation? In this context, the elevation of IL-18 may be secondary to myocardial necrosis as suggested by Seta et al.,^9^ because during the process of myocardial necrosis and repair, the activation of the inflammatory system occurs, including macrophages. Another limitation of this study is related to the sample, which in addition to being small, was obtained by convenience, which in itself can be a source of bias. The control group can be a problem, as it was selected from patients submitted to coronary angiography, who had no evidence of obstructive angiographic lesion (we recall that, for some reason, they were submitted to an invasive procedure). The coronary angiography has limitations in diagnosing atherosclerosis,^10^ because in its initial phase, there is a positive remodeling effect (Glagov effect); plaques with 70% of the total area of the vessel, may present on the angiography as a lesion smaller than 30%.^11^ Therefore, the coronary angiography should not be used as the gold standard to rule out atherosclerosis. Moreover, it is known that the plaques that most often result in ACS are mild and inflamed plaques.^5^ These limitations could explain the higher levels (although not statistically significant) of IL-18 in the control group, when compared to patients with stable coronary disease in this study (663.25 pg/mL ± 993.93 versus 353.81 pg/mL ± 273.65, respectively, p = NS).

Should IL-18 be blocked for primary prevention? Or should post-infarction ventricular remodeling be improved? Given the complexity of the immune system, and its importance in containing infectious processes and in the cell damage-repair system, its blocking might have consequences. In the case of IL-18, which is part of the caspase system, responsible for fighting intracellular infections, we could observe an increase in infections such as tuberculosis. In the Cantos (Antiinflammatory Therapy with Canakinumab for Atherosclerosis Disease) study, interleukin 1-b blocking was associated with a slight increase in infection rates (not significant), arthritis and fatal cancer (statistically significant).^12^ Regarding remodeling, the results are conflicting; interleukin 1b blocking with anakinra was promising,^13^ but the blocking of tumor necrosis factor gamma increased mortality. In experimental models, blocking IL-18 has shown to be promising in improving post-infarction remodeling.^14^

Despite the initial enthusiasm for the results of using colchicine and canakinumab, the net benefits are still limited.^12 , 15^ Therefore, it is necessary to investigate other inflammatory pathways. We still have more questions than answers: Which pathway should be blocked? When (primary or secondary prevention)? And at what intensity and for how long?
